# Effects of gyroid lattice relative density on wear and mechanical performance of 3D printed TPU and flexible nylon for footwear

**DOI:** 10.1016/j.isci.2026.115868

**Published:** 2026-04-22

**Authors:** Jing Li, Imjoo Jung, Chenhong Lang, Sunhee Lee

**Affiliations:** 1 College of Textiles and Apparel, Quanzhou Normal University, Quanzhou, China; 2 College of Textile Science and Engineering, Zhejiang Sci-Tech University, Hangzhou, Zhejiang, China; 3 Department of Fashion Design, Dong-A University, Busan, Korea

**Keywords:** Applied sciences, Engineering, Manufacturing

## Abstract

This study investigates the effect of gyroid lattice relative density on the tribological and mechanical behavior of thermoplastic elastomers made by material extrusion (MEX) for footwear applications. Three commercial filaments—lightweight foamed expanded thermoplastic polyurethane (eTPU), thermoplastic polyurethane (TPU), and polyether block amide (PEBA)—were printed as gyroid-structured cubes with infill densities of 30%, 40%, 50%, and 70%, using a nozzle temperature of 220 °C and printing speed of 50 mm/s. PEBA exhibited the most gyroid architecture. PEBA at 70% density showed the lowest wear rate of 0.33%. These findings provide a scientific foundation for tailoring the mechanical, tribological, and thermal properties of MEX-printed elastomers, offering valuable insights for optimizing outsole design and improving the performance of footwear products.

## Highlights

• Gyroid lattice density affects the mechanical and wear performance of MEX elastomers
• PEBA shows superior structural fidelity and stable gyroid architecture
• Higher infill density improves wear resistance; 70% gives best results
• PEBA at 70% density achieves the lowest wear rate, ideal for footwear outsoles

## Graphical abstract

**Figure:**
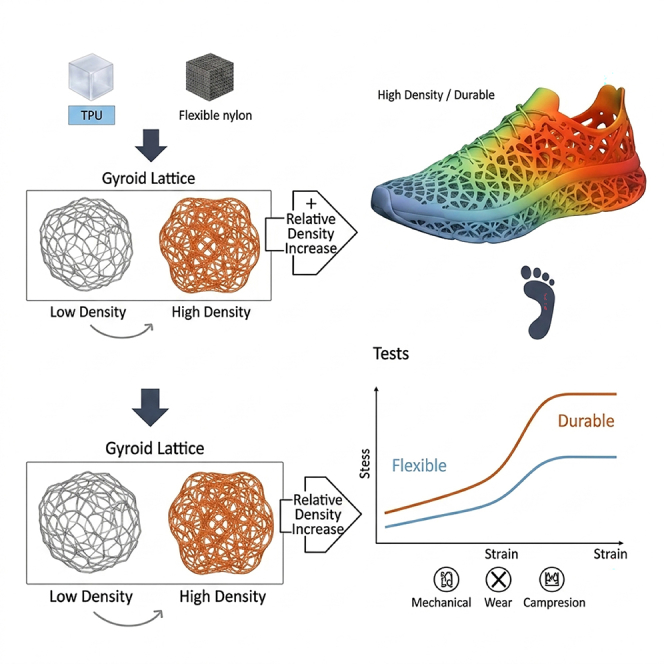

## Introduction

In recent years, the rapid advancement of material extrusion (MEX) additive manufacturing technology has provided new opportunities for innovation in footwear design and production. With its ability to create complex geometries, enable personalized customization, accelerate product development, and reduce mold-related costs, MEX has gradually become a promising alternative to traditional manufacturing approaches.^1^ Nevertheless, when compared with conventional injection molding, MEX-produced footwear components continue to face several challenges, including insufficient interlayer bonding strength, poor surface quality, and limited wear resistance.^2^ These shortcomings highlight the necessity of improving material resilience, abrasion resistance, and overall functional performance. In particular, the optimization of infill geometry and density has emerged as a key research direction to enhance footwear comfort, energy return efficiency, and durability.^3^

In footwear additive manufacturing, thermoplastic polyurethane (TPU) remains the most widely used elastomer, owing to its excellent flexibility, wear resistance, and processing stability.^4^^,^^5^ Although MEX-printed TPU exhibits good elongation and resilience, its mechanical strength is generally lower than that of injection-molded parts.^6^^,^^7^ To overcome these limitations, expanded thermoplastic polyurethane (eTPU), produced by incorporating foamed microspheres, has gained increasing attention. ETPU demonstrates a unique combination of lightweight cushioning, high rebound efficiency, and long-term durability across a broad temperature range −40°C–120 °C.^6^^,^^8^ Previous studies have reported that ETPU outperforms conventional TPU in energy return, shock absorption, and compression set resistance, making it particularly suitable for midsole and cushioning applications in performance footwear.^8^ However, challenges such as processing complexity, cell uniformity control, and integration with 3D printing processes still require further investigation.^9^

In recent years, MEX technology has been increasingly applied in the field of polymers and polymer composites. Studies have shown that printing parameters, material composition, and internal structure significantly affect the mechanical performance of printed parts. Previous research^10^ has indicated that adjusting extrusion temperature, printing speed, and infill strategy can improve interlayer bonding, thereby enhancing mechanical properties. However, these studies have mostly focused on single-material systems or cross-sectional comparisons under different experimental conditions, limiting their applicability for practical material selection in engineering applications.

Beyond traditional mechanical performance, the tribological behavior of MEX -printed parts has also attracted growing attention. Most existing studies focus on rigid thermoplastics under simple loading conditions, while the tribological response of elastomeric materials under complex loading remains underexplored.^11^ In real applications such as shoe midsoles, materials are subjected to both cyclic compression and relative sliding, and their frictional behavior is closely related to internal structural deformation and energy dissipation. Nevertheless, the current literature^12^ lacks systematic comparisons of different elastomeric materials under the same printing parameters and infill structures, which limits the engineering guidance for designing cushioning and damping structures using MEX elastomers.

Another promising material is polyether block amide (PEBA), a thermoplastic elastomer characterized by low density, outstanding elasticity, and high flexibility at low temperatures. PEBA-based midsoles have been shown to reduce running energy cost by approximately 1.88%, underscoring their potential to enhance athletic performance.^13^ Compared with TPU, PEBA generally provides superior fatigue resistance, better cold-temperature performance, and enhanced rebound, making it attractive for high-performance sports footwear.^14^ Nevertheless, its relatively high cost, limited availability in filament form, and processing challenges within MEX-based additive manufacturing remain barriers to widespread adoption.

Taken together, while TPU remains the dominant elastomer in footwear additive manufacturing due to its balance of processability and performance,^15^ both ETPU and PEBA are emerging as advanced alternatives. ETPU offers lightweight cushioning and excellent energy return, whereas PEBA provides superior resilience and low-temperature elasticity. Future research, while TPU remains dominant in 3D printing applications, both ETPU and PEBA are gaining increasing attention for high-resilience, lightweight, and long-term elasticity applications.^6^

In this context, advanced elastomeric materials such as PEBA, TPU, and eTPU have attracted attention due to their unique thermal, rheological, and mechanical properties.^16^ Although previous studies have analyzed the mechanical performance and printability of each material, they often focus on single-material systems or independent processing conditions. Direct, systematic comparisons under identical MEX parameters and gyroid infill structures are scarce.^17^ Conducting such multi-material comparisons can reveal differences in compression cushioning and frictional performance, providing a reliable experimental basis for material selection and structural optimization in shoe midsoles. Meanwhile, as MEX-printed components transition from prototypes to functional parts, conventional geometric and surface measurements are insufficient to fully capture the structure-performance relationship. Recent studies^18^ have applied multi-scale characterization combining porosity distribution, internal topology, and mechanical response, along with experimental design and inverse modeling approaches, to precisely quantify performance differences among materials under the same structural conditions. Such techniques not only improve the comparability of test results but also provide critical support for systematic analysis of multi-material MEX components.

The Gyroid, a triply periodic minimal surface without straight lines or mirror symmetry, occurs in both nanoscale and biological structures. In footwear, compression performance is crucial for comfort and cushioning, and structural optimization can enhance energy absorption and deformation uniformity. Previous studies have shown that Gyroid structures display bending-dominated deformation, smooth compression curves, and uniform plastic deformation.^19^ Incorporating twisting designs further improves their elastic modulus, stability, and energy absorption capacity.^20^ Compared to conventional lattice structures, Gyroid designs can achieve a 25–40% increase in specific energy absorption,^21^ making them highly suitable for cushioning applications in footwear. Together, these three studies show that^10^^,^^11^^,^^22^ that MEX printing parameters, especially packing density, not only determine mechanical properties, but also profoundly affect the tribological behavior of polymers and their composites. From conventional thermoplastic materials (ABS, PP) to flexible and reinforcing materials (TPU, CF composites), the research gradually moves from performance characterization to structural regulation and parameter optimization-oriented tribological design.

Recent studies have demonstrated the potential of 3D-printed TPU-based lattice and TPMS structures for cushioning and energy absorption. For instance, Holmes et al.^23^ investigated the compressive response of gyroid structures with varying relative densities, Beloshenko et al.^24^ analyzed the effect of topology and cut direction on the mechanical performance of TPU lattices, and Tilley et al.^25^ proposed functionally graded gyroid structures for enhanced cushioning. While these works provide valuable insights into design strategies and structural optimization, they primarily emphasize topology and static mechanical behavior.

However, limited attention has been paid to systematic material-level comparisons of elastomeric materials used in additive manufacturing, particularly involving TPU, expanded TPU (eTPU), and other high-performance elastomers. In addition, the linkage between fundamental material properties (e.g., thermal, rheological, mechanical, and tribological characteristics) and their practical implications for footwear midsoles remains insufficiently understood.

To address this gap, the present study conducts a comprehensive evaluation of multiple elastomeric materials fabricated via MEX, aiming to provide both scientific insights and application-oriented guidance for the design of 3D-printed sports footwear midsoles.

**Figure 1:**
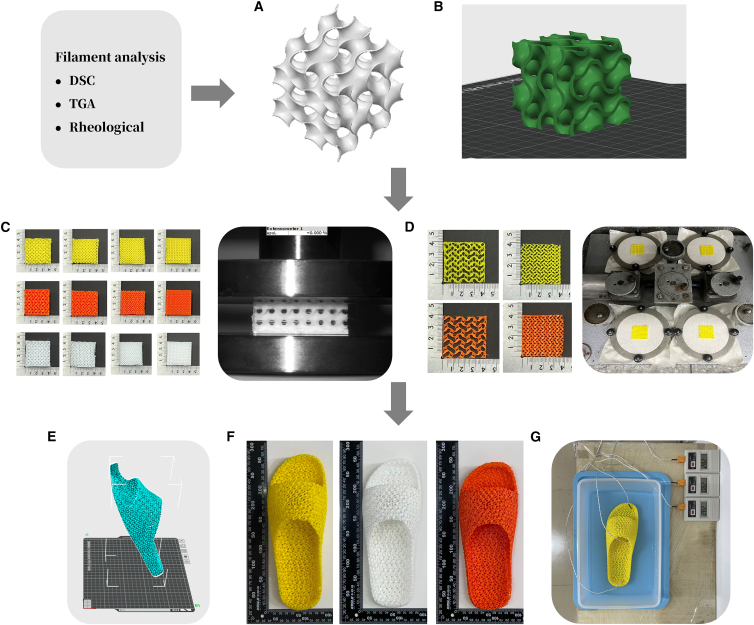
Experimental workflow for gyroid lattice structures and 3D-printed footwear (A) Modeling of gyroid lattice cubes. (B) Slicing of a gyroid-structured cube. (C) Compression testing. (D) Tribological testing. (E) Modeling of 3D-printed footwear. (F) Thermal testing setup. (G) Heat dissipation system under controlled conditions.

Specifically, this study systematically investigates the effects of gyroid infill density on the mechanical and tribological performance of three elastomeric materials—TPU, eTPU, and PEBA—fabricated via MEX 3D printing, see Table 1. The main objectives are as follows, see Figure 1.

• The thermal stability, viscoelastic behavior, and processability for three types of elastomeric filaments were confirmed through thermal properties and rheology.
• To evaluate the influence of gyroid infill density on the mechanical and tribological properties of elastomeric materials fabricated by fused deposition modeling.
• To compare the performance characteristics of eTPU, TPU, and PEBA under identical gyroid structural conditions.
• The findings of this research are expected to provide deeper insights into the relationship between 3D-printed microstructures and material performance, supporting the development of lightweight, durable, and comfortable footwear products.

**Table 1:** Specifications of the 3D printing filaments used in this study

Material	eTPU	TPU	PEBA
Supplier	Esun (Shenzhen, China)	Rosh (Guangzhou, China)	Xinbo Chuan (Shenzhen, China)
Density (g/cm^3^)	1.21	1.21	∼1.02
Melt Flow Index (MFI)	1.2 g/10min (190°C/2.16 kg)	15–25 g/10min (190°C)	Limited data
Glass transition temperature (Tg)	−9°C	∼−70°C to −107°C	∼−50°C to −60°C
Hardness (Shore A)	95	95	85
Key Features	Ultra-light density (∼100–200 kg/m^3^)	Good flexibility and wear resistance	High elasticity and high resilience

## Results

### Thermal properties of the three types of filament materials

#### Differential scanning calorimetry (DSC) analysis

**Figure 2:**
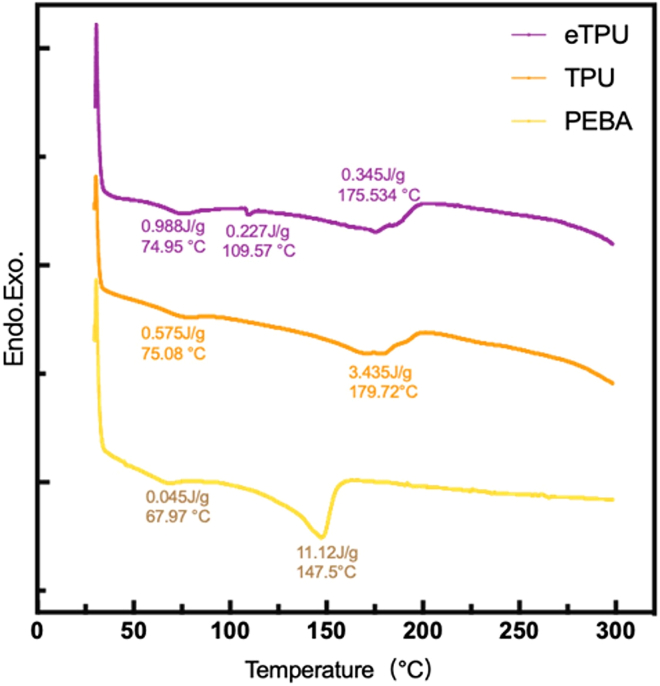
DSC curves of the three types of filament materials

The differential scanning calorimetry curves of eTPU, TPU, and PEBA filaments are shown in Figure 2. Distinct endothermic transitions were observed for each material. PEBA exhibited a sharp melting peak at approximately 147.5 °C with a high enthalpy of 11.12 J/g as strong crystallinity. A weak transition near 68 °C was also detected, which represented the thermal behavior of the soft segments. TPU presented two melting peaks at 76.08°C and 178.72 °C; the latter peak displayed an enthalpy of 3.44 J/g. These thermal transitions represented a morphology in which the hard and soft segments were separate, a characteristic behavior of segmented polyurethanes. The eTPU displayed multiple low-intensity peaks at 74.95 °C with an enthalpy of 0.388 J/g, at 109.57 °C with an enthalpy of 0.227 J/g, and at 175.63 °C with an enthalpy of 0.345 J/g, which describe the heterogeneous and partially foamed microstructure of the materials.

#### Thermogravimetric analysis (TGA) analysis

**Figure 3:**
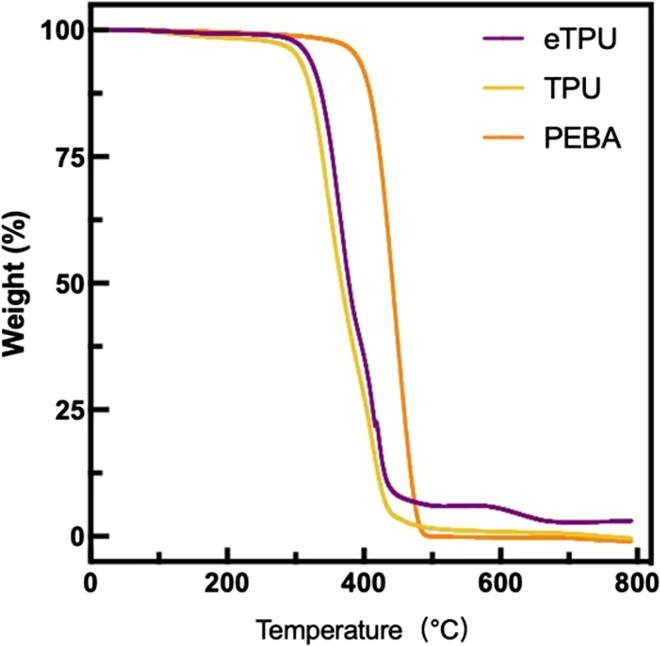
TGA curves of the three types of filament materials

Figure 3 shows the thermogravimetric analysis curves of the three filaments. All filaments underwent major weight loss above 300°C. eTPU initiated thermal degradation at a similar onset temperature of approximately 310 °C, with the main mass loss observed between 310°C and 440°C. TPU showed an earlier onset of degradation, beginning near 300 °C, followed by a primary decomposition stage spanning from 300°C to 440 °C. PEBA exhibited the highest thermal stability, with an onset decomposition temperature near 380 °C and a major weight loss occurring between 380°C and 486°C. In addition, eTPU displayed a secondary weight loss stage in the temperature range of 587°C–673°C, which was not observed for TPU or PEBA. Following thermal exposure up to 800 °C, no residual mass was observed for TPU and PEBA, while eTPU exhibited a residual mass of 3.05%. The presence of this char residue is attributed to additives or foaming agents incorporated in the eTPU formulation, which contribute to residual structural stability at elevated temperatures.

### Rheological properties of the three types of filament materials

**Figure 4:**
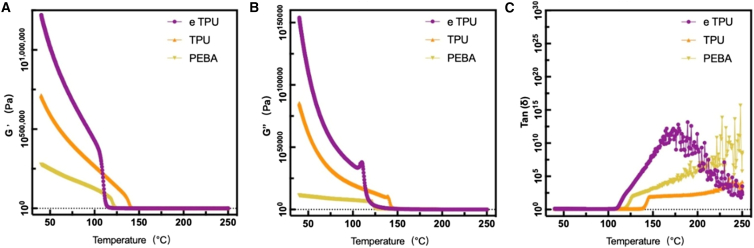
Rheological properties of filament materials (A) Storage modulus (G′). (B) Loss modulus (G″). (C) Loss factor (tan δ) of ETPU, TPU, and PEBA filaments as a function of frequency. Measurements were conducted under identical testing conditions.

Rheological measurements were performed to evaluate the viscoelastic behavior of eTPU, TPU, and PEBA. The storage modulus (G′), loss modulus (G″), and damping factor (tan δ) are presented in Figure 4 and show a clear temperature dependence. eTPU exhibited the highest G′ and G″ values across the entire temperature range of 50°C–250°C, indicating a higher modulus compared with the other materials. TPU displayed moderate G′ values with a relatively stable temperature response, whereas PEBA showed the lowest modulus values but the highest tan δ, suggesting superior damping capability and flexibility.

### Mechanical properties of gyroid-structured cubes with various infill densities and materials

#### Compressive property

**Figure 5:**
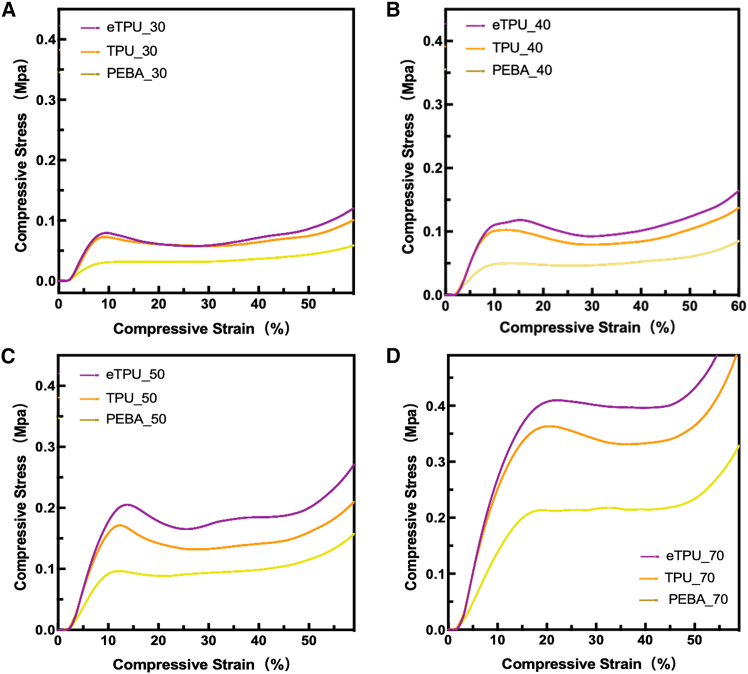
Compressive stress-strain behavior of gyroid lattice structures Stress-strain curves of gyroid-structured cubes fabricated with ETPU, TPU, and PEBA at different infill densities: (A) 30%, (B) 40%, (C) 50%, and (D) 70%.

The results of compressive stress-strain of 3D printed gyroid lattice cubes, designed in CAD with specified unit cell sizes and relative densities of 30%, 40%, 50%, and 70%, are presented in Figure 5. It should be noted that the lattice geometry was determined by the CAD model, while the 3D printing infill setting was only used to realize the designed lattice structure. The stress-strain behavior clearly reflects the influence of relative density on mechanical performance: higher-density lattices exhibited increased stiffness and load-bearing capacity, and eTPU consistently showed a superior compressive response compared to TPU and PEBA across all densities.

At a density of 30%, all materials exhibited a distinct plateau region between 10 and 30% strain, which reflected efficient absorption of compressive energy during the early stage of deformation. The eTPU sample reached the highest compressive stress, approximately 0.13 MPa, while TPU and PEBA followed in order. Also, eTPU responded with a sharper stress rise and greater recovery capability, whereas PEBA produced the smoothest and lowest curve, implying excellent ductility and flexibility under load. When the density increased to 40%, a gradual enhancement in compressive response was observed in all samples. Both eTPU and TPU displayed two-step deformation behavior that involved an initial elastic plateau followed by a slow stress build-up beyond 40% strain. The stress level of eTPU became clearly higher than that of TPU, accompanied by earlier densification. PEBA remained the softest among the three, retaining its cushioning ability throughout the compression range. At 50% density, the stiffness of all materials increased noticeably. eTPU reached its highest stress near 0.3 MPa, followed by a steady increase after 40% strain, which reflected efficient energy recovery under large deformation. TPU presented a comparable trend but with slightly lower stress amplitude, while PEBA maintained a flexible response with moderate stress growth. The separation in mechanical performance among the three materials became more apparent at this density level. At 70% density, all samples underwent a rapid stress increase beyond 50 to 60% strain as densification began. The eTPU specimen attained the steepest slope and the highest compressive stress, around 0.45 MPa, representing a strong capacity to bear mechanical loads. TPU produced a lower stress curve compared with eTPU, and PEBA retained a delayed densification response, maintaining its soft and cushioning characteristics. The overall trend across densities revealed that higher structural density enhanced both stiffness and load resistance, with eTPU consistently exhibiting superior mechanical performance compared to TPU and PEBA.

#### Tribological property

Table S1 presents microscopic images of the gyroid-structured cubes after 0, 100, and 200 abrasion cycles and Table 2 summarizes the corresponding mass loss data. As the infill density increased, surface damage decreased in all materials. Microscopic observations reveal progressive surface damage with increasing abrasion cycles, with more pronounced fibrillation and material removal in TPU compared to PEBA. Representative images of eTPU, TPU, and PEBA samples with low (30%) and high (70%) infill densities under increasing abrasion cycles (200 cycles). Images were acquired at low (×5) and high (×16) magnifications to illustrate both macroscopic deformation and microscale surface damage.

**Table 2:** Mass change and mass loss rate of gyroid-structured cubes during tribological property

Sample	Before (g)	SD	After (g)	SD	Volume (V, cm^3^)	SD	Mass loss Rate(%)
eTPU-30	0.614	±0.005	0.606	±0.004	0.074	±0.003	1.30
eTPU-40	0.685	±0.006	0.672	±0.005	0.104	±0.004	1.90∗
eTPU-50	0.633	±0.005	0.621	±0.004	0.080	±0.003	1.90∗
eTPU-70	0.788	±0.007	0.706	±0.006	0.148	±0.006	1.94∗
TPU-30	0.829	±0.006	0.805	±0.005	0.093	±0.004	2.89∗∗
TPU-40	0.845	±0.007	0.821	±0.006	0.094	±0.004	2.84∗∗
TPU-50	0.934	±0.008	0.911	±0.007	0.101	±0.005	2.46∗∗
TPU-70	1.138	±0.009	1.107	±0.008	0.126	±0.006	2.72∗∗
PEBA-30	0.627	±0.004	0.623	±0.004	0.092	±0.003	0.64
PEBA-40	0.632	±0.004	0.629	±0.003	0.095	±0.003	0.48
PEBA-50	0.696	±0.005	0.693	±0.004	0.119	±0.004	0.43
PEBA-70	0.909	±0.006	0.906	±0.005	0.080	±0.004	0.33

Notes: Data are presented as mean ± SD. eTPU: expanded thermoplastic polyurethane; Statistical analysis was performed using two-way ANOVA.∗*p* < 0.05 and ∗∗*p* < 0.01 indicate statistically significant differences.*n* = 3 technical replicates per group.

For eTPU, the mass before testing ranged from 0.614 g at 30% to 1.107 g at 70%. After repeated abrasion, the remaining mass was 0.606 g, 0.672 g, 0.621 g, and 1.107 g, corresponding to mass-loss rates of 1.30%, 1.90%, 1.90%, and 1.94%, respectively. Although the structural surfaces showed reduced peeling at higher density, the mass-loss values remained within a similar range across densities. TPU exhibited the greatest changes among all materials. The initial mass values (0.829 g, 0.845 g, 0.934 g, and 1.138 g) decreased to 0.805 g, 0.821 g, 0.911 g, and 1.107 g after testing, resulting in mass-loss rates of 2.89%, 2.84%, 2.46%, and 2.72. These values were consistently higher than those of eTPU and PEBA, and the microscopic images revealed substantial flake detachment and enlarged debris particles regardless of density. PEBA displayed the smallest reduction in mass. At 30 to 70% infill density, its initial mass ranged from 0.627 g to 0.909 g, decreasing slightly to 0.623 g, 0.629 g, 0.693 g, and 0.906 g. The corresponding mass-loss rates were 0.64%, 0.48%, 0.43%, and 0.33%, the lowest among all materials. Surface observations confirmed minimal particle formation and the most stable structural retention, particularly at 50 and 70% infill density.

One-way analysis of variance (ANOVA) was performed to compare the mass-loss rates among the three materials (TPU, eTPU, and PEBA), with material type as the independent variable and mass-loss rate as the dependent variable. Mass-loss data across all infill densities were included in the analysis. Post-hoc comparisons were conducted using Tukey’s test. Results indicated a highly significant effect of material type on mass loss (*p* < 0.001). The average mass-loss rates (mean ± SD) were 0.47 ± 0.12% for PEBA, 1.76 ± 0.31% for eTPU, and 2.73 ± 0.19% for TPU, confirming significantly higher abrasion resistance of PEBA compared with the other materials. Overall, both microscopic and quantitative assessments confirmed that PEBA maintained the highest structural durability, eTPU demonstrated moderate resistance to material loss, and TPU showed the greatest susceptibility to degradation under repeated abrasion cycles.

### Thermal validation footwear thermal test

**Figure 6:**
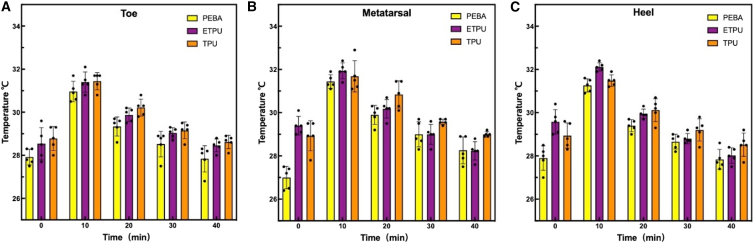
Temperature distribution in 3D-printed footwear Temperature changes measured at different regions of the shoe: (A) toe zone, (B) metatarsal zone, and (C) heel zone under controlled testing conditions.

Temperature variation at the toe, metatarsal, and heel zones was recorded for 40 min. Figure 6 presents the temporal temperature changes at 10 min intervals for each region, while representative external thermal distributions are summarized (Table S2).

Across all regions, an initial temperature increase was observed within the first 10 min, followed by gradual stabilization toward the end of the measurement period. PEBA consistently exhibited the smallest temperature rise across the toe, metatarsal, and heel zones, with a maximum increase of approximately 2.5°C–3.0 °C. The temperature of PEBA samples decreased rapidly after the peak, reaching an average surface temperature of approximately 28.2 °C at 40 min. The eTPU showed intermediate thermal behavior. Peak temperatures ranged between 31°C and 32 °C, with moderate heat accumulation observed in the metatarsal and heel zones. By the end of the test, the average surface temperature stabilized near 28.4 °C. The TPU displayed the highest temperature levels throughout the test duration. Pronounced heat accumulation was observed particularly in the heel and metatarsal regions, and elevated temperatures persisted longer than in the other materials. After 40 min, TPU samples maintained an average surface temperature of approximately 28.6 °C.

**Figure 7:**
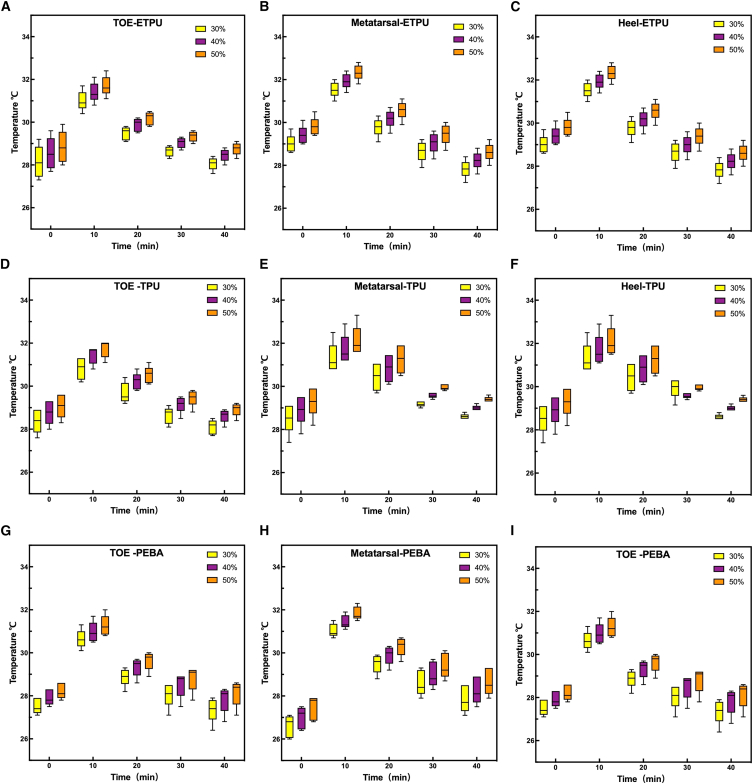
Temperature variation across different materials and foot regions Boxplots showing temperature distributions of ETPU, TPU, and PEBA in the toe (TOE), metatarsal (MET), and heel (HEEL) regions over time at different infill densities (30%, 40%, and 50%): (A–C) ETPU, (D–F) TPU, and (G–I) PEBA.

Figure 7 shows the temperature changes of the toe (TOE), metatarsal (Metatarsal), and heel (Heel) over time at different densities (30%, 40%, 50%) for different sole materials (ETPU, TPU, PEBA). The results are as follows.

#### Temperature change trend

The foot temperature of the three materials first increased and then stabilized with the increase of exercise time at all densities. The temperature peaks around 10 min after the start of exercise, then slowly decreases or remains stable. There are differences in temperature in different areas of the foot: metatarsal temperature is the highest, followed by heel, and toes are the lowest, indicating that the metatarsal area accumulates the most heat.

#### The effect of density on heat dissipation performance

Comparing sole materials of different densities, it is found that the lower the density, the lower the temperature peak, the slower the heating rate, and the better the heat dissipation performance. Low density (30%): Lowest temperature peak, slow temperature rise, and best heat dissipation performance. Medium density (40%): the peak temperature is slightly above 30%, and the heat dissipation performance is moderate; High density (50%): The highest temperature peak, the fastest temperature rise, and the worst heat dissipation performance. This law applies to all three materials and all areas of the foot, indicating that density is an important factor affecting the heat dissipation performance of the sole.

#### Material-to-material differences

The temperature change trend of ETPU, TPU, and PEBA is similar, but PEBA has a slightly larger temperature increase and relatively slow heat dissipation under high-density conditions. The temperature of the metatarsal region of TPU decreased significantly in the middle and late stages. The overall temperature of the ETPU changes smoothly. All things considered, low-density PEBA and ETPU materials are slightly better than TPU in terms of heat dissipation performance.

The quantitative values derived from infrared measurements are summarized in (Table S2). Thermal images obtained using infrared imaging further supported these observations, showing distinct spatial temperature distributions on the external surfaces of the shoes. PEBA exhibited more uniform and cooler surface patterns, while TPU showed localized regions of higher temperature retention.

## Discussion

### Comparison of thermal stability

The thermal behaviors of eTPU, TPU, and PEBA demonstrated that each material fulfills different functional roles in 3D-printed insole applications. The DSC and TGA results together clarify how crystallinity, segmental structure, and thermal resistance govern their suitability for structural stability or comfort-oriented performance.

ETPU showed a thermally dispersed response with low melting enthalpy, reflecting limited crystalline development and a heterogeneous structure associated with foaming. This thermal profile aligned with high elasticity and reduced heat-driven shape control, while the slightly higher residue observed in TGA suggested a degree of structural persistence at elevated temperatures linked to additives or foaming agents.^26^ Such characteristics favor cushioning-focused designs rather than thermally rigid components. TPU exhibited a more defined thermal response derived from its segmented hard-soft domain architecture. Although the melting peak temperature aligned with values reported for conventional TPU systems, the lower enthalpy indicated restricted hard-segment ordering, likely influenced by processing history.^27^^,^^28^^,^^29^^,^^30^ This combination supported dimensional reliability during printing while maintaining flexibility, positioning TPU as a balanced material between structural support and comfort. PEBA presented the most thermally robust behavior among the three materials. Its high melting enthalpy and delayed thermal degradation reflected strong crystalline organization and superior resistance to thermal deformation.^31^ These properties supported stable performance under high thermal loads, though reduced elastic adaptability may limit comfort when used without structural modification.

Overall, PEBA offered the highest thermal and dimensional stability, TPU provided a balanced thermal-mechanical profile, and eTPU prioritized elasticity and cushioning with moderate thermal resistance. The combined DSC and TGA analyzed therefore provides a clear framework for material selection based on targeted insole functionality.

### Viscoelastic behavior and processability

The rheological results highlight distinct temperature-dependent viscoelastic responses among the three elastomeric materials under identical oscillatory shear conditions. eTPU showed higher storage and loss modulus values across the examined temperature range from 50°C to 250 °C, corresponding to a comparatively stable elastic and viscous response under increasing temperature. TPU exhibited moderate modulus values with a relatively gradual temperature dependence, while PEBA presented lower storage and loss modulus values but higher tan δ values across the measured range, indicating an increased viscous contribution to the overall response under the applied conditions.

These differences reflect material-dependent viscoelastic behavior during temperature variation rather than intrinsic material superiority. From a processing perspective, the comparatively higher modulus response of eTPU suggests increased resistance to deformation during heating, which may influence flow behavior during extrusion-based processing. TPU demonstrates a balanced response between elastic and viscous components, whereas PEBA shows a more pronounced viscous response at elevated temperatures. It should be noted that these interpretations are limited to comparative trends observed during temperature sweep tests conducted at a fixed frequency and do not represent absolute viscoelastic property measurements.

### Mechanical performance and energy absorption

The mechanical performance was analyzed by compressive property. As a result of compressive S-S curves, at 30% density, all gyroid structured cubes showed a plateau region between 10 and 30% strain, reflecting early energy absorption. eTPU reached the highest compressive stress until 0.13 MPa, followed by TPU and PEBA. As density increased, stress and stiffness rose correspondingly. At 70% density, eTPU achieved the steepest stress increase, more than 0.45 MPa, after 50% compression, while PEBA remained the most compliant.

And the compressive behavior of gyroid structured cubes according to various materials and infill density v-strain diagrams is shown in (Table S3). The compressive properties by infill density can confirm differences in compressive behavior. At 30% density, all samples exhibited early and extensive cell wall buckling. The lattice structure began to collapse as early as 20% strain, and by 60–80% strain, the cubes showed substantial lateral expansion and loss of structural integrity. The deformation was dominated by bending and localized collapse of thin gyroid surfaces, reflecting the low stiffness associated with sparse cell networks. At 40% density, the deformation remained progressive but showed improved structural retention. Buckling was still observed at relatively low strain, but the onset of full structural collapse was delayed compared with the 30% cubes. The gyroid surfaces sustained more uniform compression, producing a smoother flattening behavior through 60–80% strain. At 50% density, the cubes maintained their shape significantly longer, and the collapse became more stable and symmetric. Early-stage deformation (0–40% strain) was characterized by uniform compression of cell walls rather than localized buckling. Even at 80% strain, the lattice preserved its overall boundary shape, suggesting increased stiffness and resistance to catastrophic collapse. The cubes with 70% density demonstrated the most robust behavior. Minimal deformation was observed at 20% strain, and noticeable buckling did not occur until higher strain levels. The structure remained highly stable throughout the compression sequence, and even at 80% strain, the gyroid architecture exhibited strong resistance to densification. This behavior reflects the high load-bearing capability derived from its thickened cell walls and reduced porosity. Overall, increasing infill density enhanced structural stability, delayed the onset of buckling, and reduced the extent of lateral deformation. While material type showed only minor visual differences in deformation mode, infill density played a dominant role in governing the mechanical response of gyroid lattice cubes under compression. These deformation characteristics, combined with the stress-strain responses, illustrate how infill density and material composition simultaneously influence compressive performance. Based on these observations, the mechanical behavior of the printed gyroid structures can be compared with findings from previous studies.

These results confirmed that the mechanical response of 3D-printed gyroid structures depended strongly on both material composition and internal density. The expanded cellular network of eTPU provided improved energy absorption and elastic recovery, while TPU maintained a balanced combination of strength and compliance. PEBA exhibited the greatest flexibility, offering deformation characteristics that are advantageous for applications requiring softness and comfort. Consistent with previous reports, micro-foamed TPU-based elastomers show enhanced compressive modulus and impact resistance, contributing to superior mechanical durability in additively manufactured components.^32^

Previous studies have also demonstrated that 3D printing parameters can significantly influence hardness and internal bonding strength.^33^ PEBA materials are known for excellent impact and creep resistance,^34^ characteristics that not only improve the performance of blended systems such as PBAT but also reduce shrinkage during fabrication. Therefore, integrating the intrinsic elasticity of PEBA with the structural advantages of gyroid architectures may further enhance deformation resistance and energy return, presenting a promising strategy for developing high-performance 3D-printed elastomeric components.

In the present study, three specimens were tested for each material and infill density condition in the compression experiments. Due to the time-consuming fabrication process and material limitations associated with multi-material 3D printing of gyroid lattice structures, producing five specimens for each condition was not feasible within the scope of this work.

Although all specimens were fabricated using the same printing orientation to ensure consistency and comparability, this approach does not eliminate anisotropy, which is an inherent characteristic of MEX-based additive manufacturing.

### Wear resistance and durability

As infill density increased, TPU samples showed a reduction in surface damage after tribological testing, but their overall resistance to wear remained lower than that of eTPU and PEBA. Low-density TPU samples at 30% exhibited large flake-type peeling, curled edges, and a high amount of coarse debris after testing. In medium density samples between 40 and 50%, the affected area was reduced to some extent, but edge breakage and particle accumulation were still clearly observed. In high density samples at 70%, the edge structure appeared relatively more intact. However, rough surface bands and large debris particles were still distributed across the worn area. Optical microscopy confirmed that, although edge integrity improved with increasing density, the size of debris particles in TPU remained relatively large, demonstrating that the improvement in surface condition with density increase was limited.

For PEBA, the abrasion rate generally decreased as density increased, reaching the lowest value at 50% density, followed by a slight increase at higher density. Low-density PEBA samples at 30% showed a large amount of powder-like and flocculent debris along the edges, with loosely attached surface layers. Medium density samples between 40% and 50% exhibited clearly reduced debris and finer particles, while high density samples at 70% maintained clear edge profiles with minimal particle adhesion. These observations show that PEBA samples maintained a more stable surface condition at medium to high densities under the applied test conditions. The eTPU samples exhibited intermediate wear behavior between TPU and PEBA. Mass loss values ranged from approximately 1.30 to 1.94%, and surface observations revealed smaller debris particles compared with TPU, although localized peeling remained visible at lower densities. Across the tested density range, the variation in mass loss for eTPU was relatively small, reflecting consistent surface degradation behavior during repeated mechanical contact. TPU, particularly in FDM applications, is generally recognized for its excellent elasticity and wear resistance; however, its actual friction and wear performance is strongly influenced by formulation variables (e.g., hardness, molecular weight), moisture absorption, and printing parameters.^35^ PEBA is widely used in footwear, where it demonstrates excellent resilience and durability, with some studies comparing its long-term performance and wear characteristics to ethylene-vinyl acetate EVA.^36^ Nevertheless, dedicated tribological data on PEBA remain scarce, and most available studies focus on injection-molded or extruded samples.

Overall, infill density played a significant role in the surface degradation behavior of all tested materials. Higher density was associated with reduced visible damage and improved continuity of the gyroid-structured cubes. Differences among eTPU, TPU, and PEBA were observed in both mass loss values and surface morphology. The present discussion is limited to comparative observations based on optical microscopy and mass loss measurements, and does not address detailed wear mechanisms or subsurface damage evolution. Taken together, the results demonst were rate a clear trend in abrasion resistance, with PEBA showing the lowest material loss, followed by eTPU and TPU. These findings confirm that both material type and infill density influence the durability of 3D printed gyroid-structured cubes under repeated abrasion contact.

### Analysis of thermal validation

The observed thermal responses followed a consistent order of TPU, eTPU, and PEBA in terms of heat accumulation. After 40 min, the stabilization temperatures of approximately 28.6 °C for TPU, 28.4 °C for ETPU, and 28.2 °C for PEBA highlight relative differences in thermal response trends under controlled conditions. It should be noted that the absolute temperature differences among materials observed in this test were relatively small, within a narrow range of approximately 0.2°C–0.4 °C across regions and time points.

PEBA exhibited the most effective thermal management among the tested materials. The limited temperature rise and rapid stabilization observed across the toe, metatarsal, and heel zones align with the known characteristics of PEBA foams, which combine low density, high elasticity, and thermal insulation properties. The segmented structure composed of polyether and polyamide domains supports low thermal conductivity on the order of 0.05 W m^−1^ K^−1^, contributing to stable thermal behavior during prolonged use.^37^ Similar thermal responses of PEBA-based footwear materials have been reported in previous studies focusing on heat transfer and comfort performance.^37^ The eTPU demonstrated intermediate thermal behavior between PEBA and TPU. Its closed-cell microcellular structure restricted excessive heat accumulation while allowing heat release through elastic deformation under thermal and mechanical loading. This structural feature promotes internal airflow and limits localized temperature concentration, as reported for microcellular elastomeric systems used in footwear applications.^38^ The observed temperature stabilization near 28.4 °C reflected a balance between cushioning performance and thermal regulation. About TPU, it showed the highest and most persistent surface temperatures, particularly in load-bearing regions. The dense polymer network and low porosity of TPU limit air circulation and delay heat diffusion, resulting in sustained thermal retention. Comparable thermal lag effects have been reported for TPU-based footwear components, where heat dissipation is slower than in foamed elastomer systems.^39^^,^^40^

Overall, the thermal performance of the tested materials is closely associated with their microstructural morphology and intrinsic thermal conductivity, consistent with previous comparative analyses of polymer-based footwear materials.^41^ From a design perspective, PEBA was well-suited for forefoot and metatarsal regions where thermal comfort is critical. And eTPU provided an effective balance between cushioning and ventilation, making it suitable for the midsole. For TPU, it offered structural stability for heel regions when combined with design strategies such as ventilation channels or hybrid material configurations. Thus, a multi-material midsole configuration incorporating PEBA in the forefoot, eTPU in the midsole, and TPU in the heel can provide a balanced combination of thermal comfort, cushioning, and structural stability, consistent with contemporary performance footwear design systems that emphasize both comfort and functional efficiency.

The results of this study show that different densities of sole materials have a significant effect on foot temperature under the same sports conditions, reflecting the important role of material density on heat dissipation performance.

#### The effect of density on temperature and heat dissipation performance

The data show that the low-density (30%) sole material has the lowest temperature peak, a slower heating rate, and the best heat dissipation performance. High-density (50%) materials have the highest temperature peak, fast heating rate, and worst heat dissipation performance. The possible reasons are: low-density materials have higher porosity, better air conductivity, and heat is easy to dissipate outward; High-density materials have a large heat capacity, accumulate heat quickly, and heat dissipation is blocked, resulting in faster temperature rise. This pattern is consistent across the three materials (ETPU, TPU, PEBA) and all areas of the foot, indicating that density is a key factor affecting temperature control in sole design.

#### Differences between materials and foot area effects

The overall temperature trends of the three materials are similar, but PEBA warms up slightly more at high densities, which may be related to its low thermal conductivity and dense material structure. There are significant differences in temperature in different areas of the foot: the metatarsal region is the highest, the toes are the lowest, and the heel is centered. This is related to the distribution of foot stress and blood circulation, and the metatarsal area is subjected to more pressure and friction during movement, generating more heat, and the heat dissipation efficiency of the material is more critical in this area.

For sole design, reducing the material density can effectively improve heat dissipation, reduce foot heat accumulation, and improve wearing comfort, especially suitable for sports shoes and shoe design in high-temperature environments. Different materials should be chosen for both mechanical and thermal properties: While low-density PEBA and ETPU dissipate heat better, their mechanical strength and durability may be compromised, so practical use needs to be considered.

The density of the sole material significantly affects the heat dissipation performance. The temperature rise of low-density materials is slow, and the heat dissipation efficiency is high, while the temperature rise of high-density materials is fast, and the heat dissipation is poor. The metatarsal region has the highest temperature and needs to be considered in the design. The research results provide a theoretical basis for the selection of sole materials and the optimization of sports shoe comfort.

### Summary

This study investigated the effects of gyroid lattice relative density and material type on the mechanical, tribological, and thermal responses of MEX-printed elastomeric structures, with a focus on footwear-related applications. The results demonstrate that increasing infill density generally enhances compressive stiffness while reducing surface degradation across all tested materials under the present experimental conditions.

Thermal and rheological analyses revealed clear material-dependent differences in crystallinity, viscoelastic behavior, and thermal response. PEBA exhibited higher thermal stability and lower temperature variation during controlled thermal testing, while eTPU and TPU showed distinct mechanical and viscoelastic characteristics associated with their respective microstructures. In terms of compression performance, eTPU exhibited pronounced cushioning behavior characterized by a plateau region, while TPU showed higher stiffness with reduced deformation capacity. PEBA demonstrated relatively uniform compressive responses across the tested density range. In the tribological evaluation, PEBA showed the lowest material loss, followed by eTPU, whereas TPU exhibited comparatively higher wear under the applied test conditions. Increasing infill density reduced material loss in PEBA and eTPU, while the wear behavior of TPU showed limited sensitivity to density variation.

From a structural perspective, these findings indicate that infill density plays a critical role in modulating the trade-off between deformation behavior and surface durability in PEBA and eTPU systems within the investigated parameter space. However, these observations remain specific to the geometries and testing configurations employed in this study. Additional factors, including manufacturing cost, scalability, and application-specific design optimization, were not quantitatively addressed and require further investigation.

Overall, the results emphasize the importance of synergistic optimization of material selection and lattice architecture in tailoring the mechanical, thermal, and tribological performance of elastomeric structures. From an application standpoint, such tunability is particularly relevant for the design of high-performance and customized footwear midsoles, where achieving a balance between cushioning, structural support, and wear resistance is essential. These findings provide a framework for rational design of lattice-based elastomeric systems in functional applications.

### Limitations of the study

This study provides a preliminary evaluation of 3D-printed elastomeric structures for footwear midsole applications, with the following limitations:

First, this study provides a preliminary evaluation of 3D-printed elastomeric structures for footwear midsole applications; however, several limitations should be acknowledged. The test specimens were based on simplified geometries, which may not fully capture the complex stress distribution and deformation behavior of real midsoles. In addition, all experiments were conducted under controlled laboratory conditions, and the potential influence of environmental factors such as temperature and humidity was not systematically considered.

Second, all samples were fabricated using a single MEX printer with fixed printing parameters and a single printing orientation. Consequently, the effects of process variability and printing-induced anisotropy were not investigated. Moreover, all materials were obtained from a single supplier and production batch, which may limit the broader applicability of the results. Although the present study provides comprehensive experimental insights into the thermal, mechanical, and tribological behavior of 3D-printed gyroid structures, several methodological limitations should be acknowledged. It should be noted that this study focuses on experimental characterization under laboratory conditions. Limitations include the relatively small number of specimens per condition and the absence of finite element analysis (FEA). Future work will expand the sample size and integrate computational modeling to further validate the findings.

Finally, the mechanical evaluation focused primarily on static or quasi-static loading conditions, while dynamic fatigue, impact loading, and long-term durability were not assessed. It should be noted, however, that the use of experimental characterization on additively manufactured, foamed elastomers offers direct relevance to real-world applications, especially for materials exhibiting nonlinear viscoelasticity, large deformation, and complex microcellular architectures, where accurate constitutive modeling remains challenging. Future work may integrate multi-scale finite element modeling with experimentally derived material parameters to further elucidate localized mechanical responses and thermo-mechanical coupling effects. Such an approach would enable predictive optimization of lattice topology, infill density, and material distribution for application-specific footwear design. The influence of build orientation-induced anisotropy was not explored, as all specimens were printed in a single orientation.

Although the mechanical and tribological properties of the 3D-printed Gyroid structures were examined, the biocompatibility of the materials was not evaluated and remains to be addressed in future work. Similarly, although the selected materials (eTPU, TPU, and PEBA) are widely used in footwear and related applications, formal biocompatibility testing according to relevant standards (e.g., ISO 10993) was not conducted in this study. Future work prior to product implementation will address both the aforementioned mechanical limitations and include necessary cytotoxicity or skin-contact safety assessments to ensure a comprehensive evaluation for practical human use.

Future work will focus on the following directions: systematically investigating the effects of printing parameters and orientation on mechanical and tribological behavior, with particular emphasis on anisotropy; introducing more realistic midsole geometries and loading conditions (including dynamic fatigue and impact); examining the long-term durability of materials under varying environmental conditions; conducting biocompatibility assessments to ensure safety for skin contact; and integrating numerical simulation and finite element analysis to enhance predictive capability and guide the design optimization of 3D-printed structures for footwear applications.

## Resource availability

### Lead contact

Requests for further information and resources should be directed to and will be fulfilled by the lead contact, Jing Li (lijing2021@qztc.edu.cn).

### Materials availability

This study did not generate new unique materials or reagents.

### Data and code availability

The data supporting the findings of this study are available from the corresponding author upon reasonable request. The datasets generated and analyzed during this study are publicly available in the Zenodo repository at: https://zenodo.org/records/18149651. All raw data, processed data, and analysis scripts used in this study are available from the corresponding author upon reasonable request.

## Acknowledgments

This research was supported by the Fujian Provincial Department of Science and Technology, grant no. 2025I0024, Fujian Provincial Natural Science Foundation (FNSF).

Special thanks are extended to the Basic Research Laboratory from Dong-A University for providing experimental facilities and technical assistance. The authors also acknowledge the contributions of colleagues and collaborators whose insights greatly enriched this work.

## Author contributions

First Authors: J.L.; Corresponding Authors: J.L and S.L. J.L. and S.L. designed the research. J.L. performed conceptualization, methodology development, and wrote the original draft. I.J. conducted the investigation and formal analysis. C.L. contributed to formal analysis, data curation, and visualization. S.L. supervised the study and was responsible for writing – review and editing. All authors participated in discussion, data interpretation, and manuscript revision, and all authors read and approved the final manuscript.

## Declaration of interests

The authors declare no competing interests.

## Declaration of generative AI and AI-assisted technologies in the writing process

During the preparation of this work, the authors used a generative AI-assisted tool solely to improve language clarity and readability. The authors reviewed and edited all content and take full responsibility for the final manuscript.

## STAR★Methods

### Key resources table

**Table:** 

REAGENT or RESOURCE	SOURCE	IDENTIFIER
**Chemicals, peptides, and recombinant proteins**		

eTPU filament	Esun (Shenzhen, China)	Density 1.21 g/cm^3^; pre-dried before 3D printing
TPU filament	Rosh (Guangzhou, China)	Density 1.21 g/cm^3^; pre-dried before 3D printing
PEBA filament	Xinbo Chuan (Shenzhen, China)	Density 1.02 g/cm^3^; pre-dried before 3D printing

**Deposited data**		

Raw data of deposited	Zenodo	https://doi.org/10.5281/zenodo.18149651

**Software and algorithms**		

Cura slicing software	Ultimaker	https://ultimaker.com/software/ultimaker-cura
GraphPad Prism	GraphPad Software, USA	Version 10
IBM SPSS Statistics	IBM	https://www.ibm.com

**Other**		

Material extrusion 3D printer	Bambu Lab	P1S
Universal testing machine	Shimadzu	AGS-X;ISO 844:2014
Differential scanning calorimeter(DSC)	TA Instruments	DSC25;ASTM E1356
Thermogravimetric analyzer (TGA)	TA Instruments	Q50; ASTM E1131
Rheometer	Anton Paar	MCR 102e
Tribological tester	Testrite Ltd. (UK)	WT37;KS M ISO 12947
Microscope	Nextecvision (Korea)	NTZ-6000
Thermal camera	Teledyne FLIR	FLIR E50
Digital thermometer	Yintiod	TM-902C

### Experimental model and study participant details

This study does not involve the use of human participants, animals, cell lines, or other biological experimental models.

### Method details

#### Materials

Three commercially available thermoplastic elastomer filaments were used in this study to fabricate gyroid-structured cubes via Material Extrusion (MEX) 3D printing process. Three types of thermoplastic elastomer filaments were used. The lightweight eTPU (Esun, China) was a thermoplastic polyurethane filament with reduced density and enhanced elasticity, suitable for lightweight footwear components. TPU (Rosh, China) was a standard thermoplastic polyurethane filament widely used in footwear prototyping due to its flexibility and durability. PEBA (Xinbo chuan, China) was a high-performance polyether block amide filament featuring superior resilience, low-temperature flexibility, and excellent abrasion resistance. All filaments were stored in a sealed and low-humidity environment at 25 ± 2 °C and under RH below 40% for at least 48 h before printing to prevent moisture absorption which could otherwise cause printing defects and degraded mechanical properties. The specification of filaments for 3D printing are shown in Table 1. According to the literature, TPU has good biocompatibility and is used in some medical applications, while PEBA has potential biocompatibility and has been applied in medical devices. However, no publicly available data confirming the biocompatibility of ETPU. No specific dermatological tests were performed in this study, and the materials are not certified for biocompatibility.

#### Design and fabrication of gyroid-structured samples

##### Terminology definition

To avoid ambiguity, the following terms are explicitly defined in this study. A “gyroid lattice” refers to a designed three-dimensional cellular structure based on a triply periodic minimal surface (TPMS) with continuous and smooth surfaces, characterized by parameters such as unit cell size, relative density, and the number of unit cells along each axis. “Relative density” is defined as the volume fraction of solid material within the lattice and is used to quantify porosity. In contrast, an “infill pattern” refers to the internal toolpath generated by slicing software to control material deposition during printing. These definitions are applied consistently throughout the study to distinguish between the designed lattice geometry and the slicer-generated infill pattern.

##### 3D printing workflow

The workflow Figure 1 covers 3D modeling, slicing, and printing of gyroid-structured cubes and footwear.

First, the cube shape was selected due to their simple structure, ease of modeling and slicing, and ability to maintain uniformity across different infill densities, facilitating comparative analysis of mechanical and wear properties. Cube was modeled and filaments for 3D printing were prepared (Figure 1A). The models were sliced using Cura software (Ultimaker, Netherlands) to generate G-code files, which were then printed on a MEX 3D printer (Topzhu P1S, Bambu Lab, China) (Figure 1B). Printing parameters were selected based on manufacturer recommendations and optimized through preliminary trials to ensure consistent deposition, dimensional accuracy, and structural integrity. Key settings were: 0.15 mm layer height, 50 mm/s print speed, 200 °C nozzle temperature, 60 °C bed temperature, and gyroid infill pattern.

The printing parameters were selected based on the filament manufacturer’s recommended processing window and the technical specifications of the printer used in this study, with relevant literature serving only as a reference framework. The final parameter values were adjusted and validated through preliminary trial prints to ensure stable fabrication and structural consistency under the experimental conditions. Specifically, a layer height of 0.15 mm and a printing speed of 50 mm/s were chosen to balance accuracy and stability; a gyroid infill pattern was adopted for its isotropic mechanical behavior and suitability for load-bearing structures; infill densities of 30%, 40%, 50%, and 70% were used to systematically investigate the effect of internal density; and the nozzle temperature (200 °C) and build plate temperature (60 °C) were set within the recommended range to ensure good interlayer bonding and print adhesion. After printing, the support structures were carefully removed, and the samples underwent necessary post-processing, including cleaning, light sanding, and inspection to ensure defect-free surfaces and dimensional accuracy, resulting in final models suitable for mechanical and tribological testing.

For testing, compression samples (20 × 40 × 40 mm^3^) (Figure 1C) and tribological samples (2 × 40 × 40 mm^3^) were prepared (Figure 1D). All specimens were printed with the same orientation for consistency, though anisotropy inherent to MEX printing remains. Although all specimens were fabricated using the same printing orientation to ensure consistency and comparability, this approach does not eliminate anisotropy, which is an inherent characteristic of material extrusion–based additive manufacturing. For each condition, three specimens were tested. While a larger sample size could further improve statistical power, three replicates are commonly used in preliminary studies for additive manufacturing. Variability was evaluated using mean ± standard deviation (SD) and coefficient of variation (CV).

Based on compression results, 30–50% infill density was selected for midsole design. Gyroid-structured midsoles were optimized in CAD for lightweight cushioning and fabricated into slipper-type footwear using TPU, ETPU, and PEBA filaments. After post-processing, thermal performance was evaluated under controlled conditions (Figures 1F and 1G).

#### Preparation of mechanical and tribological specimens

Two types of samples were fabricated:

Compression test specimens.

• Dimensions: 20 × 40 × 40 mm^3^

Tribological test specimens.

• Dimensions: 2 × 40 × 40 mm^3^

The thin plate configuration used for tribological testing ensured sufficient contact area and test stability.

All specimens were fabricated using the same printing orientation to maintain experimental consistency. However, anisotropy inherent to the material extrusion process cannot be completely eliminated.

#### Fabrication of 3D printed footwear

Based on the compression test results, an infill density range of 30–50% was selected for midsole design.The midsole geometry incorporated a gyroid lattice structure to achieve lightweight characteristics while maintaining adequate cushioning performance. Slipper-type footwear was fabricated using TPU, eTPU, and PEBA filaments under optimized printing parameters determined through preliminary filament characterization. After printing, supports were removed and minor surface finishing was performed before thermal validation experiments.

#### Thermal and rheological characterization

##### Differential scanning calorimetry (DSC)

Thermal analysis was conducted using a DSC25 instrument (TA Instruments, USA) under a nitrogen atmosphere, performed according to ASTM E1356. Approximately 3 mg of sample was sealed in an aluminum pan and heated from 30°C to 300 °C at a rate of 10 °C/min. The first heating cycle was used to preserve the thermal history introduced during filament production and the fused deposition modeling process.

The thermograms were analyzed to determine.

• Glass transition temperature (Tg)
• Melting temperature (Tm)
• Melting enthalpy (ΔHm)

##### Thermogravimetric analysis (TGA)

Thermal stability was analyzed using a TGA Q50 instrument (TA Instruments, USA), performed in accordance with ASTM E1131. Approximately 4 mg of sample was heated from 30°C to 800 °C at a rate of 20 °C/min under nitrogen atmosphere. Mass loss curves were recorded to evaluate thermal degradation behavior.

##### Rheological analysis

Rheological properties were measured using an Anton Paar MCR 102e rheometer with 25 mm parallel plate geometry. Temperature sweep tests were conducted from 40°C to 250 °C at 10 °C/min under oscillatory shear with a frequency of 1 Hz.

The following parameters were recorded.

• Storage modulus (G′)
• Loss modulus (G″)
• Loss factor (tan δ)

#### Compression testing

Compression tests were performed using a Shimadzu AGS-X universal testing machine equipped with a 5 kN load cell. Testing conditions.

• Crosshead speed: 15 mm/min
• Temperature: room temperature

The tests followed ISO 844:2014, which specifies compression testing methods for rubber and elastomeric foams. For each condition, three specimens were tested to evaluate the compressive stress–strain response and deformation behavior.^42^^,^^43^^,^^44^ Due to fabrication constraints, the sample number was limited; therefore, the results are intended to provide comparative insights rather than statistically validated mechanical properties.

##### Tribological testing

Tribological properties were evaluated according to KS M ISO 12947 using a WT37 tribological tester (Testrite Ltd., UK).

Testing conditions:

• Motion path: Lissajous motion.
• Sliding speed: 30 cycles/min.
• Total cycles: 200.
• Abrasive surface: 200-grit sandpaper.
• Contact diameter: 38 mm.
• Normal load: 500 g.
• Environmental conditions: 25 °C, 55% RH.

Surface morphology before and after testing was observed using a microscope (NTZ-6000, Nextecvision, Korea) at ×5 and ×16 magnifications. Abrasion volume was calculated using:

$$V=\frac{\Delta m}{\rho }$$

where Δm represents mass loss and ρ represents material density. This calculation converts mass loss into volume loss for comparative evaluation of wear performance.

#### Thermal validation of 3D printed footwear

Thermal performance was evaluated under controlled conditions to assess heat transfer behavior.

The bottom surface of the midsole was placed in contact with water maintained at 10 °C, while the upper chamber containing approximately 750 mL of water was maintained at 50 °C, representing the approximate thermal environment of a human foot.

Temperature measurements were recorded at.

• heel zone
• metatarsal zone
• toe zone

using three K-type thermocouples connected to digital thermometers (TM-902C). Measurements were recorded every minute for 40 min. Additionally, thermal images were captured using an FLIR E50 thermal camera to analyze surface temperature distribution.

### Quantification and statistical analysis

All experiments were performed in triplicate unless otherwise stated. Data are presented as mean ± standard deviation (SD). Statistical analysis was conducted using GraphPad Prism 10.

• One-way ANOVA with Tukey’s post-hoc test was applied for multiple group comparisons

Statistical significance was defined as:

p<0.05

Error bars in graphs represent one standard deviation.

### Additional resources

Raw data supporting this study publicly available. Description: Zenodo repository for raw experimental data: https://doi.org/10.5281/zenodo.18149651.
