# Supplementary material for: Effects of gyroid lattice relative density on wear and mechanical performance of 3D printed TPU and flexible nylon for footwear
Source: iScience. 2026 Apr 22;29(6):115868. doi: 10.1016/j.isci.2026.115868 (PMC13186003; doi:10.1016/j.isci.2026.115868)
Supplement: Document S1. Tables S1–S3 [file mmc1.pdf]

iScience, Volume *29*

Supplemental information

Effects of gyroid lattice relative density on wear and mechanical performance of 3D printed TPU and flexible nylon for footwear

Jing Li, Imjoo Jung, Chenhong Lang, and Sunhee Lee

SUPPLEMENTAL INFORMATION

Supplemental information can be found online at XX

Document S1. Table S1–S3.

Table S1. Abrasion behavior of lattice structures fabricated from eTPU, TPU, and PEBA at different relative densities (30%, 40%, 50%, and 70%). The first column shows the original samples, while the subsequent columns present surface morphology evolution after 0 and 200 abrasion cycles under different magnifications (×5 and ×16). – Related to **Table 1.**

Table S2. External surface temperature distributions of 3D-printed shoes made from different materials (ETPU, TPU, and PEBA) over time (0–40 min) measured by infrared thermography. – Related to **Figure 5.6.**

Table S3. Compressive deformation behavior of gyroid structured cubes with different materials (eTPU, TPU, PEBA) and infill densities (30–70%) at increasing compressive strain (0–80%). – Related to **Figure 4.**

Table S1. Abrasion behavior of lattice structures fabricated from eTPU, TPU, and PEBA at different relative densities (30%, 40%, 50%, and 70%). The first column shows the original samples, while the subsequent columns present surface morphology evolution after 0 and 200 abrasion cycles under different magnifications (×5 and ×16).

Material	Density (%)	Image of sample	Abrasion cycle (magnification)		
			0 (× 5)	200 (× 5)	200 (× 16)
eTPU	30	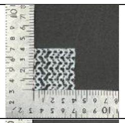	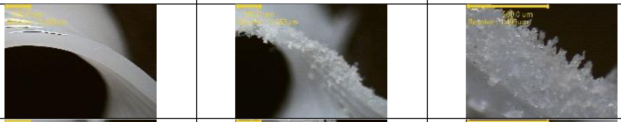		
	40	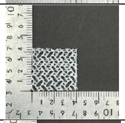	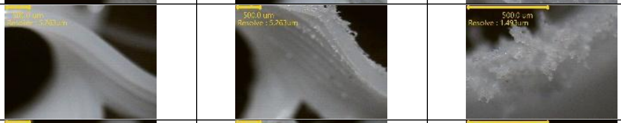		
	50	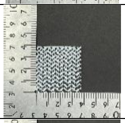	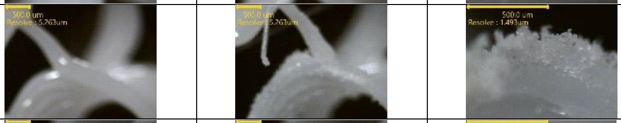		
	70	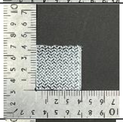	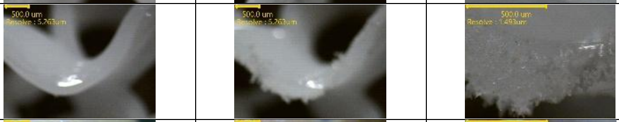		
TPU	30	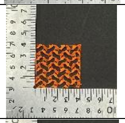	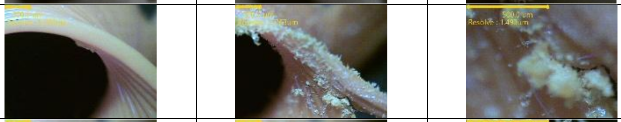		
	40	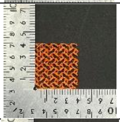	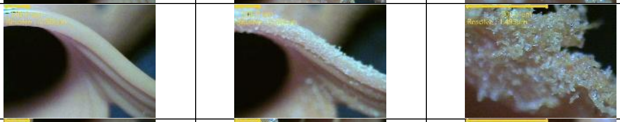		
	50	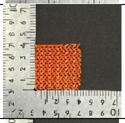	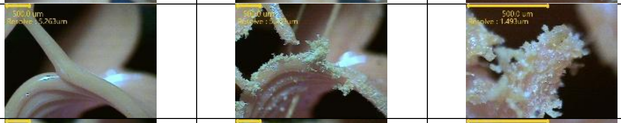		
	70	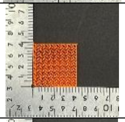	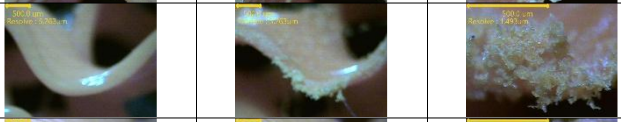		
PEBA	30	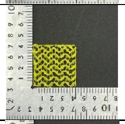	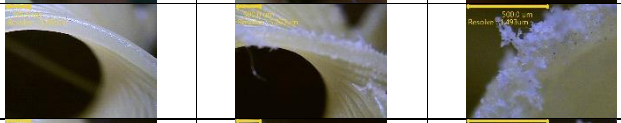		
	40	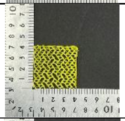	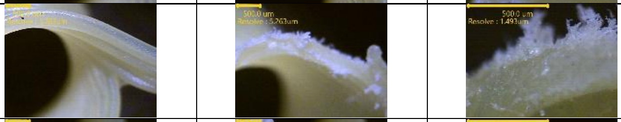		
	50	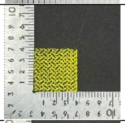	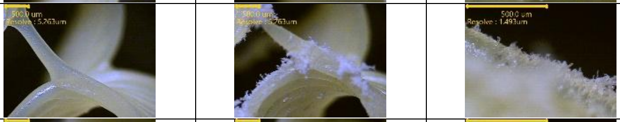		
	70	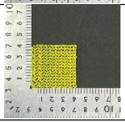	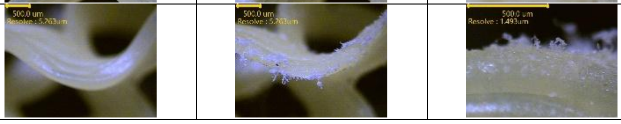		

Table S2. External surface temperature distributions of 3D-printed shoes made from different materials (ETPU, TPU, and PEBA) over time (0–40 min) measured by infrared thermography.

	Time (min)				
	0	10	20	30	40
ETPU	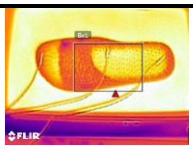	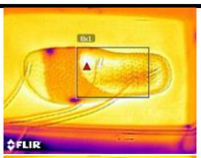	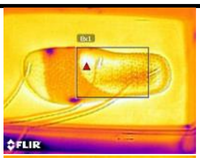	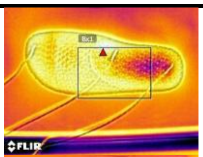	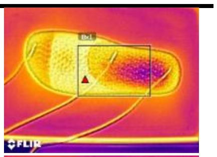
TPU	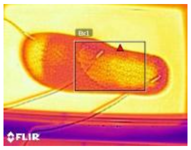	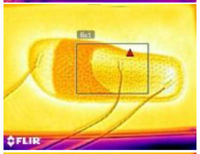	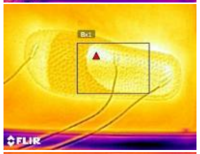	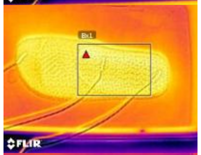	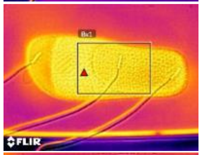
PEBA	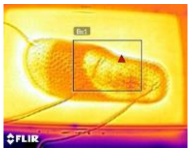	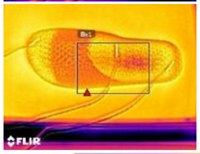	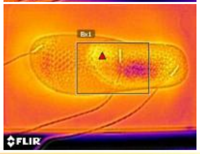	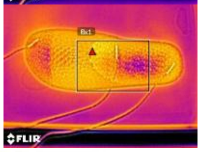	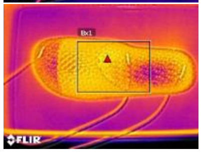
Range	0°C 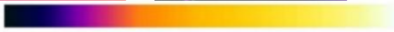 50°C				

Table S3. Compressive deformation behavior of gyroid structured cubes with different materials (eTPU, TPU, PEBA) and infill densities (30–70%) at increasing compressive strain (0–80%).

Material	Density (%)	Compressive strain (%) 0 / 20 / 40 / 60 / 80
eTPU	30	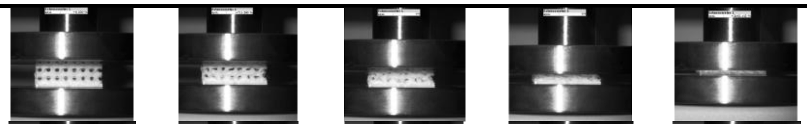
	40	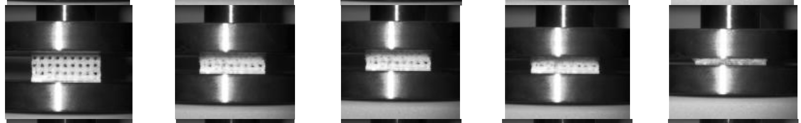
	50	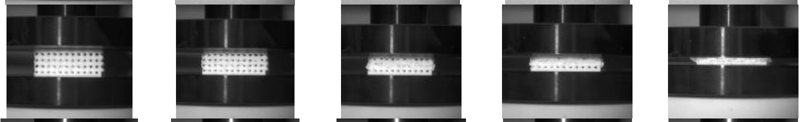
	70	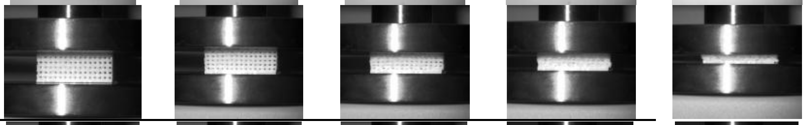
TPU	30	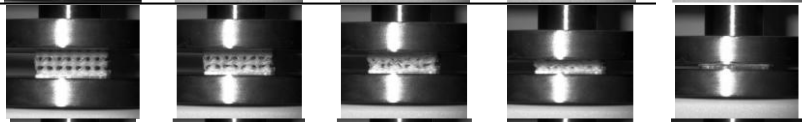
	40	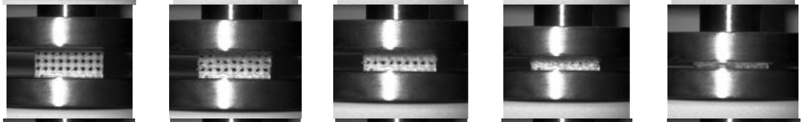
	50	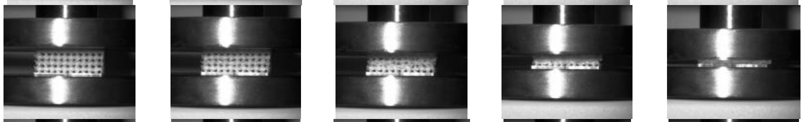
	70	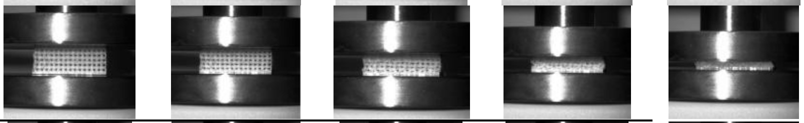
PEBA	30	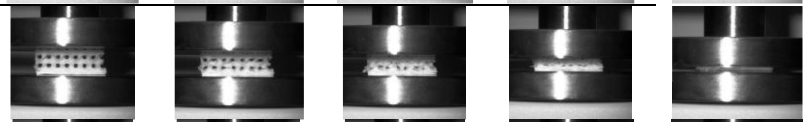
	40	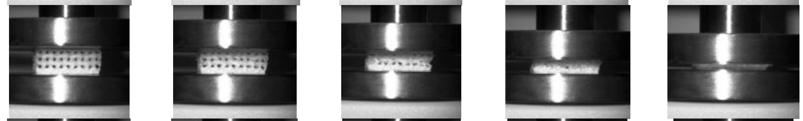
	50	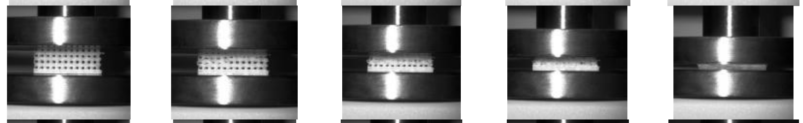
	70	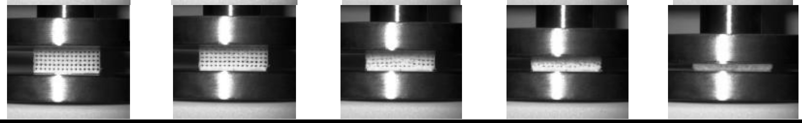
